# Does spatial attention modulate sensory memory?

**DOI:** 10.1371/journal.pone.0219504

**Published:** 2019-07-11

**Authors:** Fabiano Botta, Elisa Martín-Arévalo, Juan Lupiáñez, Paolo Bartolomeo

**Affiliations:** 1 Department of Experimental Psychology, and Brain, Mind, and Behavior Research Center (CIMCYC), University of Granada, Granada, Spain; 2 INSERM U 1127, CNRS UMR 7225, Sorbonne Université, Institut du Cerveau et de la Moelle épinière (ICM), Paris, France; University of Muenster, GERMANY

## Abstract

According to some theoretical models, information contained in visual short-term memory (VSTM) consists of two main memory stages/storages: sensory memory, a system wherein information is stored for a brief time with high detail and low resistance to visual interference, and visual working memory, a low-capacity system wherein information is protected from visual interference and maintained for longer delays. Previous studies have consistently shown a strong relationship between attention and visual working memory. However, evidence is contradictory on whether or not attention modulates the construction and maintenance of visual representations in sensory memory. Here, we examined whether and how spatial attention differentially affects sensory and working memory contents, by separately analysing attentional costs and attentional benefits. Results showed that both sensory memory and visual working memory were reliably affected by the distribution of spatial attention, suggesting that spatial attention modulates the VSTM content starting from very early stages of memory storage. Moreover, endogenously attending a specific location led to similar performance in sensory and working memory, and therefore to larger attentional benefits in working memory (where there was more room for improvement than in sensory memory, because of worse performance in unattended locations). On the other hand, exogenous attentional capture by peripheral unpredictive cues produced invariant attentional costs and invariant attentional benefits regardless of the memory type, with performance being higher in sensory memory than in working memory even at the attended location.

## Introduction

When we observe the external environment, we have the impression of experiencing a rich and detailed visual setting. Is this feeling genuine, or is it just an illusory impression? The answer to this question represents a matter of debate in the field of consciousness studies, and is directly related to the distinction made by Ned Block between two forms of consciousness: phenomenal and access consciousness. According to Block [1], phenomenal or P-consciousness represents the experiential properties of our perceptions (e.g., sensations, feelings, thoughts, wishes, and emotions), while access or A-consciousness refers to the process by which information is made available to other cognitive mechanisms (e.g., memory, reasoning, and/or decision making). According to the phenomenal overflow argument proposed by Block, the contents of our phenomenal experiences are much richer and detailed than the representations we can access at a given time [2]. In this sense, the phenomenal overflow argument states that the feeling of perceiving a rich visual experience is not an illusion: information would be available as part of our phenomenal experience, but would not be reportable.

Most of the empirical support of this theoretical argument is based on the iconic memory experiments carried out by Sperling [3]. Subjects are presented with a brief presentation of a 4x3 array of letters, and asked to report as many letters as possible from the whole array. They are usually able to report about four letters in their correct positions, despite claiming to have a strong impression of having seen all the letters. However, if participants are required to report only the letters contained in one of the rows of the array, as indicated by a spatial retro-cue presented immediately after the array offset, they can recall all of the letters presented in the cued row, again four letters. Since participants are able to recall the letters contained in any retro-cued row, Sperling suggested that all the letters presented in the uncued rows must be available right after the array presentation in a sensory memory store, but they would vanish while reporting those presented in the cued row. According to the phenomenal overflow argument, this effect would be a clear example of phenomenal consciousness without access consciousness, and for this reason the content of iconic memory is thought to be related to phenomenal consciousness. However, it is still controversial whether the large amount of information that is experienced without being accessible reflects phenomenal or unconscious processing, and, consequently, whether our impression of visual completeness is illusory or not.

The parallelism between iconic memory and phenomenal consciousness was explicitly proposed by Lamme [4]. In Lamme’s model, P- and A-consciousness are respectively associated with the two stores that, according to the traditional view, constituted visual short-term memory: the brief but highly detailed iconic memory store and the sparse but sustained memory known as visual working memory. This model stipulates that, during the first 100-150ms after stimulus onset, many visual representations are available in P-consciousness, which is implemented by local recurrent activations between the visual striate area (V1) and the visual extrastriate areas, and whose content is thought to reflect iconic memory. Subsequently, the activations in V1-V3 feed-forward to V4; as time passes, however, these activations lose strength, with a concomitant reduction of high-resolution phenomenal representations. At this later stage, the competition between representations increases, and only those engaging in global recurrent activations from visual areas to fronto-parietal regions evolve in A-consciousness, which would be related to visual working memory content. More recently, the existence of an additional memory store has been proposed. According to the multiple-store theory [5,6] this “fragile” memory, situated at an intermediate stage between iconic and visual working memory, would be a high-capacity sensory memory store like iconic memory, but with a longer duration, and independent of afterimages.

**Fig 1:**
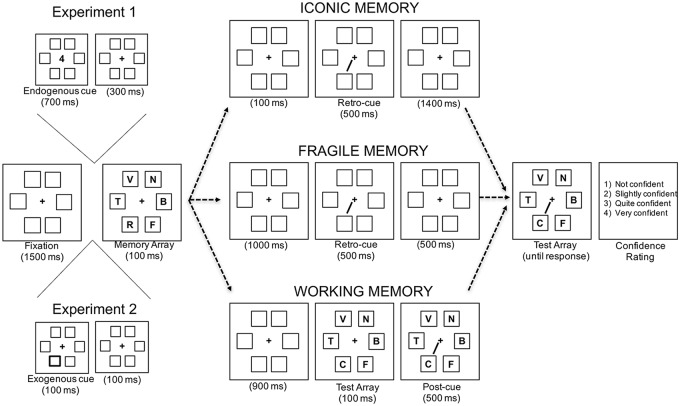
Schematic illustration of the sequence of events in a given trial. In all experiments, the three conditions of memory (iconic memory, IM; fragile memory, FM; working memory, WM) were presented. In Experiment 1, an endogenous cue consisting of a digit, appeared before the memory array and predicted the location of the probe that was subsequently signaled by the retro-cue or by the post-cue. In Experiment 2 and 3, an exogenous cue consisted of a 100-ms thickening of the contour of one of the six placeholders was instead presented before the memory array. In Experiment 2 the exogenous cue was not predictive, while in Experiment 3 it predicted the opposite location as the most likely to be probed.

Iconic, fragile, and visual working memory have been usually assessed by using a change detection paradigm along with either a retro-cue or a post-cue (see Fig 1). For instance, participants are required to decide whether the letters contained in a memory display are identical or not to those presented in a subsequent test display. A cue presented after the memory array indicates which location will be probed, and participants are instructed that only the letter indicated by the cue may change from memory to test display. The change between the memory and the test display occurs in 50% of cases. Importantly, the cue can be presented either during the blank between memory and test display (retro-cue condition), or right after the test display (post-cue condition). In the retro-cue condition, participants can access the relevant information at the probe location *before* the interfering information produced by the test display is presented. On the other hand, in the post condition, the relevant information can be accessed only after the interfering visual information has occurred. Depending on the delay between the retro-cue and the offset of the memory display, retro-cues are thought to probe iconic (short delay) or fragile memory (long delay). Post-cues are instead used to assess visual working memory. It is worth noting that even though long delay retro-cues are considered by some authors to target fragile short-term memory as a system dissociable from both iconic and working memory [5–7], this idea is not universally accepted. Many studies (e.g., see Souza & Oberauer [8] for a review) have extensively used the differences between retro-cues and post-cues to investigate how the internal focus of attention affects visual working memory representations. Note that in this case the theoretical approach is different, because retro-cues and post-cues are used to assess different processes (such as focusing of attention and visual interference) taking place in the same structure (visual working memory), rather than to assess the content of two different structures (fragile visual short-term memory and visual working memory). In the present study, we considered long delay retro-cues as assessing fragile visual short-term memory, in order to facilitate comparison with previous studies of the same kind. The issue about whether or not fragile visual-short term memory might be considered as an independent system from visual working memory will be discussed in the General Discussion.

According to the multiple-store theory, similar to iconic memory, but in contrast with visual working memory, fragile short-term memory would be easily overwritten by similar information presented at the same location as that encoded. This resistance against visual interference that would dissociate visual working memory from sensory memories would be explained by the different role exerted by attention on the three types of memories. Consistent with the above-mentioned parallelism between types of short-term memories and types of consciousness, according to Lamme [9] sensory memories/phenomenal consciousness would be attention-free, while working memory/access consciousness would require attention, thus supporting the case that attention and consciousness, or at least phenomenal consciousness, can be dissociated. This idea has been empirically supported by Vandenbroucke, Sligte, and Lamme [7], who manipulated the availability of attentional resources while participants were doing a change detection task measuring fragile and visual working memory, by increasing the temporal uncertainty about the moment in which the to-be-remembered information would be presented, or by asking participants to simultaneously perform a demanding task. In general, results showed that the reduction of attentional resources was more detrimental for working memory than for fragile memory, and this finding was interpreted as evidence supporting a dissociation between attention and sensory memory/phenomenal consciousness.

In a subsequent study, Pinto et al. [10] manipulated spatial attention before the memory array by using a precue that indicated the most likely location to be probed. The authors focused on the analysis of the attentional costs, and found that they were similar for the fragile and the working memory conditions. Pinto et al. [10] interpreted this result as evidence that only working memory needs spatial attention. However, alternative interpretations are possible, with attention having an effect in both sensory and working memory, if one considers not only the attentional costs, i.e., the decrement in performance at the unattended locations compared to a neutral location, but also the overall effect of attentional orienting, i.e., the improvement in performance at the attended location compared to the unattended locations (see General discussion).

The current study has two aims. The first was to understand whether and how spatial selective attention affects sensory memories (iconic and fragile memory). This question may seem trivial considering the huge amount of studies having shown attentional effects in perceptual tasks. However, as already observed by others [11], the role of attention on sensory memories has often been ignored, on the assumption that sensory memories were just the result of retinal stimulation, or that sensory memory and perception are overlapping. Furthermore, as stated above, some concluded that sensory memories are not modulated by attention [7]. The second aim was to assess potential differences in the attentional effects exerted on iconic, fragile, and visual working memory, as a function of whether spatial attention is oriented exogenously or endogenously. Covert orienting of spatial attention (i.e., without eye movements), indeed, is based on at least two different control mechanisms (e.g., [12,13]): endogenous orienting, in which attention is focused by means of top-down processes, and exogenous orienting, in which attention is focused by bottom-up control mechanisms based on perceptual saliency. Previous studies have shown that these two mechanisms of attentional orienting can differently affect perception [14–17], spatial conflict resolution [18], and visual working memory [19–21]. Nonetheless, it is not clear whether or not these two modes of focusing spatial attention affect in a similar way the three above-described stages of short-term memory.

To accomplish our aims, we combined a typical cost-and-benefit task with change-detection tasks assessing iconic, fragile, and visual short-term memory (Fig 1) in two separate experiments. In a first experiment, endogenous attention was manipulated by presenting, before the memory array, an endogenous spatial cue consisting of a digit indicating one of the six possible locations where the items to remember were going to appear (see [19,21] for a similar procedure). The cue was predictive about the most likely location to be probed. Experiment 2 employed peripheral spatial cues to modulate exogenous attention. Cues appeared at one of the six possible item locations, before the memory array, and were unpredictive about the probe location. The effect of spatial attention on the three types of short-term memory was measured by using both objective measures (accuracy) and subjective measures (confidence ratings). In both experiments, participants’ eye-movements were tracked to ensure that participants did not directly fixate the cued locations, with consequent contamination of the results by overt attention.

We expected to observe significant attentional effects in both sensory memories and working memory. However, we predicted that the three memory stores would be differently modulated depending on the way (endogenous or exogenous) by which attention was spatially allocated. In particular, endogenous attention might mostly improve the content of working memory, given the shared dependency on fronto-parietal networks of endogenous attention and working memory maintenance [22]. For this reason, the bias produced by endogenous spatial attention was expected to gradually increase in magnitude from the iconic storage to the fragile memory and finally to the working memory storage. However, exogenous attention should mainly facilitate sensory memories, given its prominent effect on sensory areas [23].

## Experiment 1

The aim here was to establish how endogenous attention affects the progress of the visual information from iconic to fragile and visual working memory. Before the memory array, an endogenous central symbolic cue occurred, which predicted the most likely location to be probed. Furthermore, neutral cues were used in some trials. These cues did not indicate any specific location, but were presented with the same timing as the informative (valid/invalid) cues to control for temporal preparation among conditions, and served as a baseline to assess attentional benefits and costs. Attentional effects on the three memory storages were examined by comparing the patterns of performance obtained with neutral trials, to that obtained with valid trials (attentional benefits), and invalid trials (attentional costs).

### Methods

#### Participants

The group of participants consisted of 30 healthy volunteers from the Sorbonne University in Paris, France (10 males, mean age 23 years, range 19–28 years). The experiment was conducted at the Centre Multidisciplinaire des Sciences Comportementales Sorbonne Universités-INSEAD. In this and in all the following experiments, all participants had a normal or corrected-to-normal visual acuity, normal color discrimination, and no history of neurological or psychiatric problems. They were naïve as to the purpose of the study, which lasted for approximately 60 min. All participants gave written informed consent. The experiment was approved by the ethical committee of the Centre Multidisciplinaire des Sciences Comportementales Sorbonne Universités-INSEAD, in accordance with the ethical standards of the 1964 Declaration of Helsinki.

#### Apparatus

Eye movements were recorded by using a Tobii X2-60, tracking binocularly at 60 Hz with a spatial resolution of 0.1°, and a maximum average gaze position error of 1°.

#### Stimuli

The stimuli were displayed on a light gray background on a LCD video monitor (refresh rate = 60 Hz) located in a dark and quiet room. The distance between the participant’s head and the video monitor was approximately 75 cm. A 0.75° × 0.75° fixation cross was continuously displayed at the center of the screen.

Each memory and test array consisted of 1.21° × 1.83° letters presented inside each of the six 2.3° × 2.3° placeholders (Fig 1). The placeholders were squares evenly spaced around an imaginary circle, centered at fixation, with a radius of approximately 6.2°. The letters were upper case, randomly picked from the set BCDFGHJKLNPQRSTVZ (i.e., all the consonants of the French alphabet except M and W, which were not used in order to avoid possible confusion between similar letters). The endogenous pre-cue consisted of a digit (1 to 6) presented at the centre of the screen, indicating in 62% of the trials the location that would be probed in the test array (i.e., valid trials); the remaining trials were instead invalid, and were equally distributed between all combinations of cue location × probe location. The neutral pre-cue was a “?” symbol presented at the centre. The retro-cue and the post-cue consisted of black lines (0.08 by 3.4◦ of visual angle) that pointed from the fixation cross to one of the six possible placeholders.

#### Procedure

Participants were required to maintain their gaze at the central fixation in the middle of the screen throughout each trial. Each trial began with the presentation of the six placeholders and a central red fixation cross that turned black after 500 ms. Another 1,000 ms later, either the endogenous pre-cue indicating one of the six possible placeholders, or the neutral pre-cue, which did not indicate any specific location, was presented for 300 ms. The temporal parameters (presentation durations and SOAs) of the the endogenous cue in Experiment 1 and of the exogenous cue in Experiment 2 were chosen to maximize their respective effects [13,24]. Participants were informed that each pre-cue digit was associated with a specific placeholder location, following a clockwise order. Because neutral trials represented the baseline for calculating costs and benefits, the neutral pre-cue was presented with the same temporal characteristics as the endogenous pre-cue, to control for participants’ temporal preparation to the memory array. After a 700-ms blank period following the pre-cue offset, the six letters constituting the memory array were presented for 100 ms within the placeholders. The memory array was presented for 100 ms instead of 250 ms [7] to make the task more demanding.

Participants were encouraged to attend to the location indicated by the endogenous pre-cue, because that location most likely would be the location probed by the subsequent retro- or post-cue. Participants were also asked to remember as many letters as possible. Memory for letters, rather than line orientations, was tested to minimize grouping/chunking effects [25], and to increase task difficulty [5], which probably reduces the occurrence of ceiling effects. Retro-cues and post-cues, indicating the probe location, were introduced at different latencies during the trial depending on the memory condition. The 500ms retro-cue was presented in both the iconic and the fragile memory conditions. In the iconic memory condition, the retro-cue was presented 100ms after the offset of the 100ms memory array and 1,400ms before the test array. In the fragile memory condition, it was presented 1,000ms after the offset of the 100ms memory array and 500ms before the test array (see Fig 1). The 500-ms post-cue was presented 1,000 after the offset of the memory array and 100 ms after the on-set of the test display. The delay between the memory array and the test array was 2,000 ms for the iconic and fragile memory conditions, and 900 ms for the working memory condition. Note that both the retro-cues in the fragile memory condition and the post-cues in the working memory condition were provided 1,000 ms after the memory array, in order to keep the two conditions comparable in terms of time of maintenance. The test array remained on screen until response or 5,000 ms had elapsed. Participants were required to press with their left hand one of two keys positioned on the left side of the keyboard in order to report whether or not the letter probed by the retro-cue or by the post-cue matched the letter presented at the corresponding location in the memory array. The change of the probed letter occurred on 50% for types of trials. This forced-choice response constituted the basis of the objective task performance.

After each response, participants were required to use the mouse with their right hand to click on the corresponding option to indicate how confident they were about their objective response: (1) not confident, (2) slightly confident, (3) quite confident, and (4) very confident. Confidence ratings constituted the basis of the subjective measure of the task performance.

The experiment consisted of 6 blocks of 96 trials each, for a total of 576 trials equally distributed among the three memory conditions. The endogenous pre-cue was presented in 468 trials (81.25%) and it correctly indicated the upcoming probe location (i.e., valid trials) in 62% of those trials; the remaining trials in which the endogenous pre-cue was presented were instead invalid, and were equally distributed among all combinations of pre-cue location × probed location. Concerning statistical analyses, a first main analysis compared performance on valid, neutral and invalid conditions, to assess attentional costs and benefits as a function of memory type. A second analysis considered the distance between the pre-cued location and the target position to explore how endogenous attentional distribution affects performance in the three memory conditions. For this analysis, the invalid locations adjacent to the pre-cued locations were categorized as short-distance locations, while the other three locations opposite to the cue were considered as long-distance locations (see [10,20]). The neutral pre-cue was presented in 108 trials (18.75%). All trials were randomly mixed between blocks. The participants were allowed to rest between blocks. Before the start of the experiment participants performed 2 blocks of 30 training trials each. In the first block the memory array was presented for 1,000 ms and participants received visual feedback on whether they had responded correctly or not. If necessary, the first practice block was repeated until participants fully understood how to perform the task. In the second practice block, participants were presented with trials identical to the experimental trials.

#### Data analysis

The objective performance was analysed by calculating response accuracy (percentage of correct responses). To assess how participants’ consciousness about their objective performance was affected by the manipulation of attention and memory, participants’ mean confidence ratings on correct response trials were calculated.

To determine how spatial attention affected the three memory types, a first overall analysis was performed on accuracy and on mean confidence ratings with the within-subjects factors of Attention (valid, invalid, neutral) and Memory Type (iconic, fragile, and working memory). To test our specific hypothesis, the differences between neutral and invalid trials (costs) and between attended and neutral trials (benefits) were compared between memory conditions to specifically establish whether attentional costs and benefits varied across iconic, fragile and working memory. We then performed a further additional analysis to specifically analyse the effect of endogenous attentional distribution depending on memory type with the within-subjects factor of Distance (valid, short distance, and long distance) and Memory Type (iconic, fragile, and working memory). Bonferroni correction was applied to all post-hoc comparisons.

### Results

Trials in which gaze deviated more than 2° from fixation during the memory array were discarded from the analysis. One participant was excluded for breaking fixation in more than 50% of the trials, and another one for performing at chance level (her/his performance was more than 2 standard deviations below the mean). Finally, the data file of one participant was corrupted and could not be recovered. For this reason, the final sample for the analysis was of 27 participants.

#### Accuracy

This analysis revealed a main effect of Memory Type F(2, 52) = 33.7, p<.001, η^2^ = 0.56, with higher accuracy in the iconic memory condition (M = 78.6, 95% CI = 75.1–82.1) than in the fragile memory condition (M = 74.7, 95% CI = 71.9–77.6), t(26) = 4.57, p<.001, d = 0.88, and in the fragile memory condition than in the working memory condition (M = 71.4, 95% CI = 69.-73.8), t(26) = 4.25, p<.001, d = 0.81. The main effect of Attention was also significant, F(2, 52) = 63.01, p<.001, η^2^ = 0.70, showing both significant benefits, i.e., higher accuracy on valid (M = 86.0, 95% CI = 82.3–89.7) than on neutral trials (M = 71.6, 95% CI = 68.4–74.8), t(26) = 8.83, p<.001, d = 1.70, and costs, i.e., higher accuracy on neutral than on invalid trials (M = 67.3, 95% CI = 63.9–70.7), t(26) = 3.68, p = .003, d = 0.70. Finally, we observed an interaction between Attention and Memory Type, F(4,104) = 5.43, p<.001, *η*^2^ = 0.17. To assess whether attentional costs and benefits varied across iconic, fragile, and working memory, we compared attentional costs and benefits between memory conditions.

**Fig 2:**
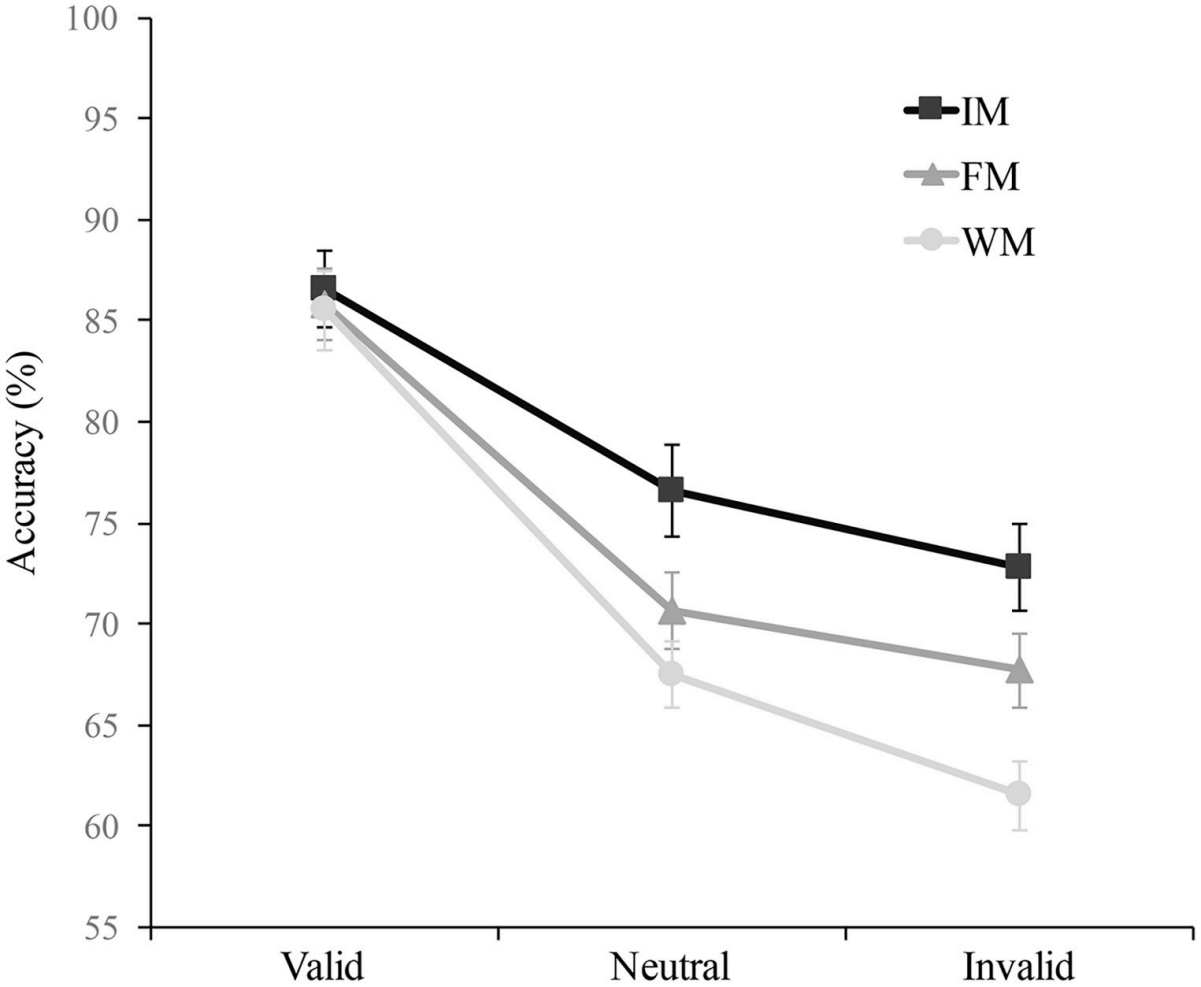
Mean accuracy (percentage of correct) of the objective task as a function of attentional condition (Valid, Neutral, Invalid) and of memory condition (iconic memory, IM; fragile memory, FM; working memory, WM) in Experiment 1. The error bars represent the standard error of the means.

This further analysis revealed that while attentional costs were comparable across the three memory types, F(2,52)<1, p = .52, *η*^2^ = 0.024, attentional benefits were not, F(2,52) = 7.86, p = .001, *η*^2^ = 0.23, being higher in working memory (M = 18, 95% CI = 13.6.3–22.3) and fragile memory (M = 15.1, 95% CI = 11.3–18.8) than in iconic memory (M = 10, 95% CI = 5.8–14.3), respectively t(26) = 3.76, p = .003, d = 0.72 and t(26) = 2.58, p = .047, d = 0.49. Note also that accuracy at the endogenously attended location was pretty identical for all memory types, all ps>.9 (see Fig 2 and Table 1).

**Table 1:** Means, standard errors and 95% confidence intervals of accuracy and confidence ratings for the two experiments, as a function of attentional condition and memory type.

		Accuracy (%)			Confidence Ratings		
		IM	FM	WM	IM	FM	WM
**Exp. 1**	Valid	86.6(0.19)	85.8(0.18)	85.5(0.2)	3.54(0.07)	3.54(0.07)	3.51(0.08)
		82.8–90.5	82.1–89.5	81.4–89.5	3.39–3.7	3.38–3.69	3.34–3.68
	Neutral	76.6(0.22)	70.7(0.18)	67.5(0.17	3.17(0.07)	3.02(0.09)	2.95(0.09)
		72.2–81.1	66.9–74.5	64.1–70.9	3.01–3.33	2.83–3.21	2.75–3.14
	Invalid	72.8(0.23)	67.7(0.19)	61.5(0.16)	2.95(0.09)	2.86(0.09)	2.63(0.09)
		68.1–77.5	63.8–71.6	58.2–64.8	2.77–3.14	2.67–3.05	2.75–3.14
	Short	75.3(0.25)	72.7(0.21)	63.6(0.22)	3.06(0.09)	3(0.09)	2.73(0.11)
	Distance	70.1–80.5	68.3–77.1	59.2–68	2.85–3.26	2.8–3.19	2.51–2.96
	Long	70.3(0.24)	62.8(0.26)	59.4(0.18)	2.85(0.09)	2.73(0.10)	2.52(0.08)
	Distance	65.2–75.3	57.5–68	55.6–63.2	2.66–3.04	2.52–2.93	2.35–2.70
**Exp. 2**	Valid	91.6(0.16)	89.2(0.17)	85(0.22)	3.55(0.09)	3.51(0.08)	3.49(0.11)
		88.2–95	85.6–92.8	80.5–89.6	3.37–3.74	3.32–3.69	3.26–3.72
	Neutral	79.2(0.17)	73.6(0.20)	71.5(0.14)	3.17(0.10)	3.09(0.10)	3.06(0.10)
		75.7–82.7	69.5–77.7	68.6–74.3	2.96–3.38	2.88–3.3	2.84–3.28
	Invalid	76.5(0.15)	70.3(0.14)	65.7(0.13)	3.11(0.09)	2.99(0.09)	2.88(0.10)
		73.5–79.6	67.5–73.2	63.1–68.4	2.91–3.30	2.79–3.19	2.67–3.09
	Short	78.8(0.16)	71.4(0.19)	65.4(0.16)	3.16(0.10)	3(0.10)	2.9(0.11)
	Distance	75.5–82.1	67.4–75.4	62.1–68.7	2.95–3.36	2.79–3.21	2.66–3.13
	Long	74.3(0.18)	69.3(0.15)	66.1(0.16)	3.06(0.09)	2.97(0.10)	2.86(0.10)
	Distance	70.6–78	66.1–72.5	62.8–69.3	2.86–3.25	2.76–3.19	2.65–3.07

Note: IM = Iconic Memory; FM = Fragile Memory; WM = Working Memory

The second analysis aimed at analysing the effect of endogenous attentional distribution and showed that accuracy was significantly modulated by the distance between the attended location and the probe location, F(2, 52) = 57.9, p<.001, η^2^ = 0.69; a trend analysis indicated a significant quadratic component, p<.015, suggesting that accuracy, rather than gradually decreasing with distance, abruptly decreased from valid to the short distance locations and then slightly further decreased from short to long distance locations where it reached the minimum. However, coherently with the first analysis, the main effect of Distance was qualified by a significant interaction with Memory type, F(2, 52) = 4.87, p = .001, η^2^ = 0.15. Post-hoc analysis revealed that the decrement of accuracy from the attended location to short distance locations was significantly higher on working memory than on both iconic and fragile memory, respectively t(26) = 3.76, p = .003, d = 0.72 and t(26) = 3.86, p = .002, d = 0.74. The decrement of accuracy from short distance to long distance was comparable in all the memory types, all ps>.36.

#### Confidence ratings

The pattern of results was pretty similar to that observed in the analysis of the accuracy. Also in this case, we observed a main effect of Memory Type F(2, 52) = 18.35, p<.001, η^2^ = 0.41, with higher mean confidence in the iconic memory condition (M = 3.22, 95% CI = 3.08–3.37) than in the fragile memory condition (M = 3.14, 95% CI = 2.98–3.29), t(26) = 3.37, p = .007, d = 0.64, and in the fragile memory condition than in the working memory condition (M = 3.03, 95% CI = 2.88–3.17), t(26) = 3.44, p = .006, d = 0.66. The main effect of Attention was also significant, F(2, 52) = 55.9, p<.001, η^2^ = 0.68, with participants being more confident on valid (M = 3.53, 95% CI = 3.37–3.68) than on neutral trials (M = 3.05, 95% CI = 2.88–3.21), t(26) = 7.8, p<.001, d = 1.5 and on neutral than on invalid trials (M = 2.81, 95% CI = 2.64–2.99), t(26) = 5.08, p<.001, d = 0.97. Finally, we observed a significant interaction between Attention and Memory Type, F(4,104) = 5.62, p<.001, *η*^2^ = 0.17. Further analysis revealed that while attentional costs did not vary across the three memory types, F(2,52) = 2.06, p = .13, *η*^2^ = 0.07, benefits were instead modulated depending on memory type following the same pattern observed in the accuracy data, F(2,52) = 5.92, p<.001, *η*^2^ = 0.18; benefits were higher in working memory (M = 0.55, 95% CI = 0.39–0.72) and fragile memory (M = 0.51, 95% CI = 0.39–0.73) than in iconic memory (M = 0.37, 95% CI = 0.22–0.51), respectively t(26) = 3.21, p = .002, d = 0.62, and t(26) = 2.52, p = .055 (marginal), d = 0.48.

The second analysis revealed that accuracy was significantly modulated by the distance between the attended location and the probe location, F(2, 52) = 54.6, p<.001, η^2^ = 0.67; a trend analysis indicated a significant quadratic component, p = .003, suggesting that participants’ confidence, rather than gradually decreasing with distance, abruptly decreased from the attended location to the short distance locations and then slightly further decreased from short to long distance locations where it was at minimum. However the main effect of Distance significantly interacted with Memory type, F(2, 52) = 5.06, p<.001, η^2^ = 0.16. Post-hoc analysis showed that the decrement of response confidence from the attended location to short distance locations was significantly higher on working memory (M = 0.77, 95% CI = 0.50–1.04) than on both iconic (M = 0.48, 95% CI = 0.32–0.65) and fragile memory (M = .26, 95% CI = 0.12–0.41), respectively t(26) = 3.15, p = .01, d = 0.60 p = .003 and t(26) = 4.01, p<.001, d = 0.77. The decrement of accuracy from short to long distance was comparable in all the memory types, all ps>.9.

## Discussion

The results of the first experiment showed that endogenous attention produced a strong significant modulation over the three types of memory. In general, the attentional modulation was characterized by an abrupt drop in accuracy between attended locations and locations near the attentional focus and by a further slight reduction of accuracy at further locations. However, this modulation affected significantly more working and fragile memory than iconic memory, and was mainly due to an increment in attentional benefits. Attentional costs were indeed almost identical in the three memory conditions.

The analysis of the subjective responses corroborated the same pattern of results observed in the analysis of the objective performance.

## Experiment 2

As previously mentioned, attention can also be focused in a stimulus-driven manner, as when it is captured by a perceptually salient object. Thus, in the second experiment, we used peripheral non-predictive cues to assess whether and how exogenous attention affects the three short-term memory stores.

### Methods

#### Participants

A different group of 30 participants (14 males, mean age 23, ranging from 18 to 29 years) from the Sorbonne University of Paris gave written informed consent to participate in the experiment. The experiment was approved by the ethical committee of the Centre Multidisciplinaire des Sciences Comportementales Sorbonne Universités-INSEAD.

#### Apparatus, stimuli, procedure and data analysis

Everything was the same as in the first experiment except for the following. The central endogenous cue was replaced by a peripheral cue, which consisted of a 100-ms thickening of the contour of one of the six placeholders in which the letters were presented (see Fig 1). The peripheral cue was presented 200 ms before the memory array, and did not predict the future probe location. The neutral cue consisted of the 100-ms thickening of the contour of all the six placeholders. Participants were instructed that the cues were irrelevant for the task and had to be ignored.

### Results

As in Experiment 1, trials in which gaze deviated more than 2° from fixation during the memory array were discarded from the analysis. Five participants were excluded because the eye movements data could not be properly recorded for technical problems. Moreover, one participant was excluded for doing the task at chance level, with performance more than 2 standard deviations below the mean. The final sample consisted of 24 participants.

#### Accuracy

**Fig 3:**
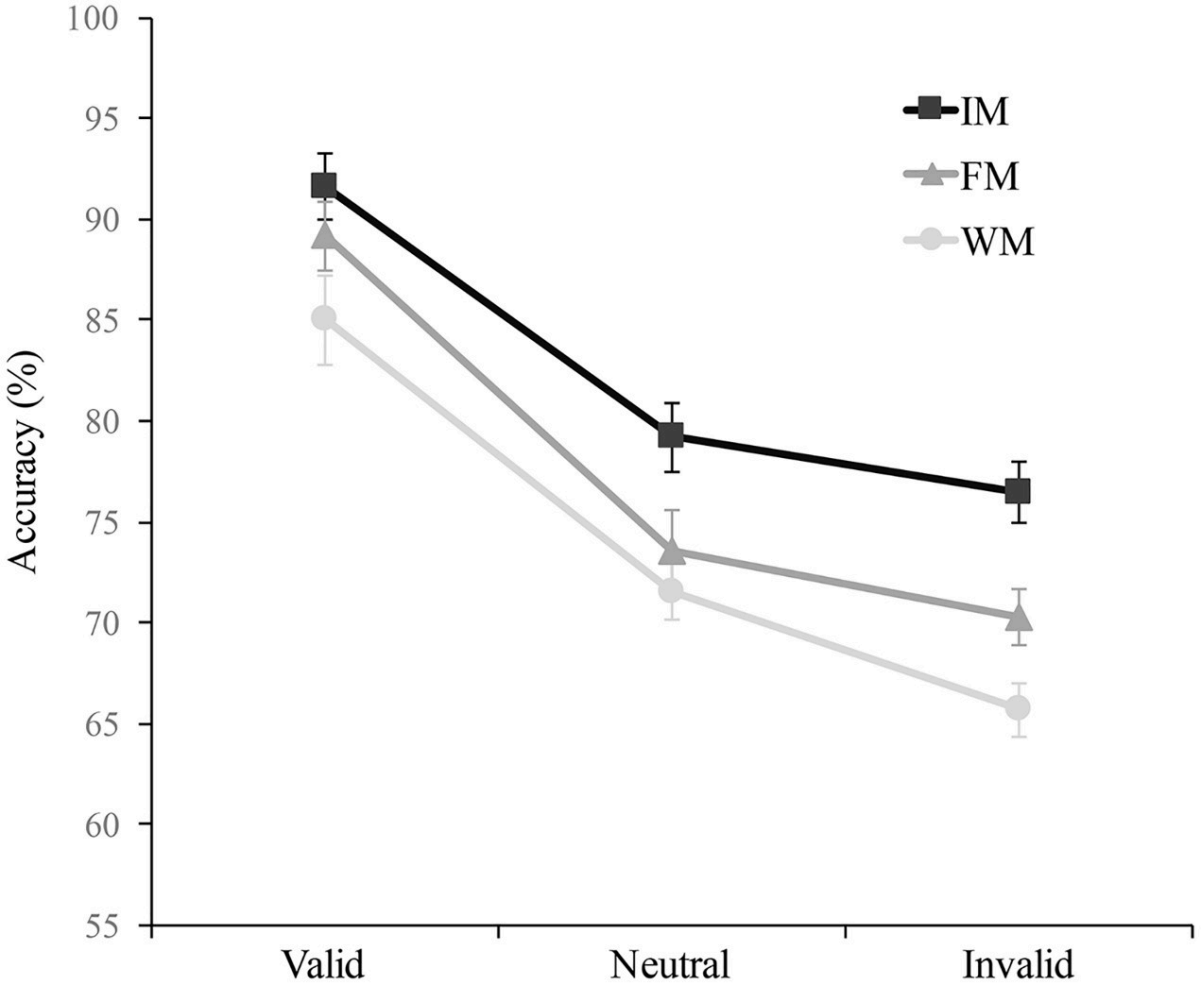
Mean accuracy (percentage of correct) of the objective task as a function of attentional condition (Valid, Neutral, Invalid) and of memory condition (iconic memory, IM; fragile memory, FM; working memory, WM) in Experiment 2. The error bars represent the standard error of the means.

A main effect of Memory Type occurred F(2, 46) = 23.06, p<.001, η^2^ = 0.50; post-hoc analysis revealed higher accuracy in the iconic memory condition (M = 82.4, 95% CI = 80.1–84.8) than in the fragile memory condition (M = 77.7, 95% CI = 75.1–80.3), t(23) = 3.98, p = .002, d = 0.81, and in the fragile memory condition than in the working memory condition (M = 74.1, 95% CI = 71.9–76.3), t(26) = 2.96, p<.02, d = 0.60. The main effect of attentional condition was also significant, F(2, 52) = 78.15, p<.001, η^2^ = 0.77, showing both attentional benefits, with higher accuracy on valid (M = 88.6, 95% CI = 85.6–91.6) than on neutral trials (M = 74.8, 95% CI = 72.5–77), t(26) = 7.96, p<.001, d = 1.62, and costs, with higher accuracy on neutral than on invalid trials (M = 70.9, 95% CI = 68.4–73.4), t(26) = 5.29, p<.001, d = 1.08. In this case, contrary to what happened in Experiment 1, the interaction between Attention and Memory Type failed to reach significance, F(4, 92) = 1.2, p = .31, *η*^2^ = 0.05, suggesting that the attentional effect was similar across memory types, or that the difference between memory types was independent of the attentional conditions (Fig 3). More specific analyses revealed that neither benefits nor costs were significantly different between memory types, respectively F(2, 46) = .68, p = .69, η^2^ = 0.02 and F(2, 46) = 1.2, p = .3, η^2^ = 0.05. Interestingly, and unlike the results of Exp. 1 (where performance in the three memory types reached the same level at the endogenously attended location), in Exp. 2 performance at exogenously attended locations was lower in working memory than in iconic memory, t(23) = 3.24, p = .01, d = 0.66. This result shows a clear effect of memory type also at the exogenous attended location, not only at the unattended locations.

The second analysis showed a significant main effect of Distance, F(2, 46) = 78.9, p<.001, η^2^ = 0.77; as in the first experiment a trend analysis revealed a significant quadratic component, p<.001, suggesting that accuracy abruptly diminished from valid to the short distance locations and then slightly further decreased from short to long distance locations where it reached the minimum. However, in line with the first analysis, the effect of Distance was not modulated depending on Memory Type, F(4, 92) = 1.88, p = .11, *η*^2^ = 0.07.

#### Confidence ratings

The first analysis revealed a main effect of Memory Type F(2, 46) = 8.59, p<.001, η^2^ = 0.27, with higher mean confidence in the iconic memory (M = 3.28, 95% CI = 3.09–3.46) condition than in both fragile memory condition (M = 3.20, 95% CI = 3.01–3.38), t(23) = 2.93, p = .02, d = 0.59, and working memory condition (M = 3.14, 95% CI = 2.95–3.34), t(23) = 3.81, p = .003, d = 0.77. The main effect of Attention also reached significance, F(2, 44) = 57.05, p<.001, η^2^ = 0.71, with higher accuracy on valid (M = 3.52, 95% CI = 3.32–3.71) than on neutral trials (M = 3.11, 95% CI = 2.91–3.31), t(23) = 7.37, p<.001, d = 1.50 and on neutral than on invalid trials (M = 2.99, 95% CI = 2.79–3.19), t(23) = 4.88, p<.001, d = .99. Finally the interaction between Attention and Memory type failed to reach significance, F(4, 92) = 1.46, p = .219, *η*^2^ = .06.

Similarly as in the objective performance, confidence ratings were significantly modulated depending on the distance between the attended location and the probe location, F(2, 46) = 52.06, p<.001, η^2^ = 0.69; again the trend analysis indicated a significant quadratic component, p<.001, suggesting that confidence ratings abruptly decreased from the exogenously attended location to the short distance locations and then slightly further decreased from short to long distance locations. The interaction between Distance and Memory Type failed to reach significance, F(4, 92) = 2.06, p = .092, *η*^2^ = .08.

#### Discussion

As in the first experiment, performance was strongly biased by attention for all the three types of memory. As in Experiment 1, the attentional bias was characterized by an abrupt decrease of accuracy from the attended location to the short distance locations, and by a further drop at long distance locations. However, in this case, differently from Experiment 1, attentional modulation was found to be similar for all memory stores. In contrast with Experiment 1, not only attentional costs but also benefits were almost identical for the three memory conditions, with no significant differences among them. To test whether the modulation of benefits in memory depended on the way in which attention was allocated, we analysed the interaction between the within-participants factors of Memory Type (Iconic, Fragile, and Working memory) and the between-subjects factor of Type of Attention (endogenous in Exp.1 vs. exogenous in Exp.2) on benefits (i.e., difference between valid and neutral trials), which failed to reach significance, F(2, 98) = 2.12, p = .12, *η*^2^ = .038. However, it is worth noting that the experiments were conceived as independent experiments and run in different moments. This could have increased variability and reduced the statistical power of this last analysis, which was presumably already low because of the between-subjects design. To further assess whether we had sufficient evidence to support that benefits were comparable in Experiment 2 across the three memory stages, we performed a Bayesian analysis comparing the attentional benefits in the three memory types. We used the default multivariate Cauchy priors (center = 0 r = 0.707) implemented in JASP, because they locate the probability mass in realistic ranges without over-represent any specific value. Also, these priors have been shown to fit a large set of psychological results with moderate effect sizes [26] to be widely applicable, and lead to Bayes factors characterized by desirable theoretical properties [27,28]. The analysis revealed a BF_10_ = 0.15, indicating substantial evidence for the null hypothesis [29] that benefits were equivalent for the three memory types. The same analysis performed in the data of Experiment 1 revealed a BF_10_ = 32.6, which is instead considered as strong evidence for the alternative hypothesis that benefits are different depending on memory type. The absence of interaction between memory and attention in Experiment 2 suggests that even when attention was supposed to be maximal (i.e., when the probe was presented at the peripherally cued location), performance decreased through the different memory stages. This is particularly interesting considering that in the first experiment performance at the endogenously attended locations was nearly identical in the three memory conditions. However, further studies combining endogenous and exogenous attentional manipulations simultaneously and/or as a within-participant factor are needed to better assess this conclusion.

## General discussion

The present study had two main aims: 1) to assess whether spatial attention biases information encoding in sensory memories (iconic and fragile) or not, and 2) to investigate whether its contribution is differently modulated by the way attention is spatially distributed (exogenous vs. endogenous) and by the memory type (sensory vs. working memory). To pursuit these goals, in two separate experiments we manipulated selective endogenous and exogenous spatial attention during the encoding phase, to assess whether and how the distribution of attentional resources, as well as the mode they were distributed, affected the three short-term memory stores.

We expected significant attentional effects in both sensory memories and working memory in both experiments. Moreover, we predicted that endogenous attention would mostly improve the efficiency of working memory, on the basis of the shared dependency on fronto-parietal networks of endogenous attention and working memory maintenance [22] while exogenous attention would mainly facilitate sensory memories, given its prominent effect on sensory regions [23]. In agreement with these predictions, we observed that selective spatial attention, independently of the mode (endogenous or exogenous) by which it was distributed during the encoding phase, reliably affected accuracy in both sensory memory (iconic and fragile) and working memory. However, the nature of the attentional effect on the three memory stores varied depending on the way by which attention was spatially allocated. In the first experiment, consistent with our predictions, we observed that when attention was endogenously oriented, the effect of attention on accuracy varied across the 3 memory stages, with attentional benefits increasing from working to fragile and from fragile to iconic memory, and attentional costs being similar for the three memory stages. On the other hand, in Experiment 2, when attention was automatically and exogenously captured by non-predictive peripheral cues, it produced similar modulatory effects (i.e. similar benefits and similar costs) on the three memory stores. This differential attentional modulation between the two experiments was likely due to a different data pattern at valid locations, which was characterized by pretty identical accuracy for the three memory types in Experiment 1, and by a significantly higher performance in iconic than in working memory, in Experiment 2.

Finally, the analysis of confidence ratings followed the same pattern observed in the analysis of accuracy, suggesting that participants were conscious about their performance, consistent with previously described strong correlations between objective and subjective performance [30]. Future studies should directly analyse how attentional mechanisms modulates metacognition in different short-term memory stages, by keeping accuracy constant among conditions [6,7,10], thus allowing a more direct analysis of attentional modulations of meta-cognition independently of objective performance.

### Does spatial attention modulates sensory memory?

A main motivation of the present study was to shed light on the existent open debate concerning the role of selective attention on sensory memory/phenomenal consciousness, with some scholars strongly supporting the idea that sensory memory, be it iconic or fragile, is attention-free [6,7,10], while others support the contrary hypothesis that even sensory memory necessarily depends on attention [11,31,32].

In our opinion, the response to this question critically depends on how the attentional modulation observed in the current study or in previous research is explained. For our results we used the logic proposed by Vandenbroucke et al. [7], according to which the lack of attention to the memory array should selectively hinder subsequent memory for attention-dependent stages. Thus, if “attention is only necessary for visual working memory, but not for fragile VSTM, performance should only decrease in the post-change cue condition” (Vandenbroucke et al. [7] pp. 1561). In the present experiments, this logic (see also Mack et al. [11,31] and Persuh et al. [32]) would have predicted the absence of attentional effects in the iconic and fragile memory conditions, in terms of attentional costs, benefits, or both. Thus, the attentional modulations that we observed for sensory memory should be interpreted as evidence that attention has a role also in this process. The fact that the effect observed in sensory memory was reduced in comparison to that observed in working memory (see the present Experiments 1 and Vandenbroucke et al. [7]), does not allow a categorical distinction between the two kinds of memory regarding the role of attention. That is, “less than” is not the same as “no” modulation.

Pinto et al. [10] proposed a different interpretation. They manipulated spatial attention in a similar way to the present Experiment 1, and concluded that attention affects working memory but not sensory (i.e., fragile) memory. However, this conclusion was based on a different logic than that used by Vandenbroucke et al. [7], with a focus on the mere attentional costs in fragile and working memory. The authors proposed that both fragile memory and working memory are available before visual interference, but only working memory is available *after* visual interference. Thus, if the attentional costs in the two memory conditions are the same, then one may conclude that sensory memory is an attention-free store separable from working memory. In other words, the attentional costs measured in the sensory memory conditions would be attributable to working memory. The fact that no extra costs were observed for sensory memory led them to conclude that it was not modulated by attention.

In our view, however, this logic of interpretation cannot be falsified for the following reason: if it is accepted that the use of a the retro-cue equally allows the access to both sensory memory (iconic and fragile) and working memory, then there is no possibility to assess fragile memory independent of working memory. The assumption made by Pinto et al. [10] is that at the moment the retro-cue is presented two kinds of representations are available: those that could possibly be masked by new interfering information (sensory memory representations) and those that would resist visual interference (working memory representations). In other words, representations that would resist visual interference would be those sensory memory representations that are endogenously attended. Those representations that would be masked without the retro-cue, are assumed to be attention-free, unless it is shown that reduction of attention towards them reduces the likelihood that they will be recalled to a greater extent than when they are masked. Moreover, it is also assumed that the attentional focusing of attention during retro-cueing leaves the sensory representations unaltered.

However, it appears difficult (if not impossible, at least in our opinion), to demonstrate that fragile memory is different from working memory on the basis of the role exerted by selective spatial attention, in the absence of an experimental condition that allows measuring its content independently and separately from the other element of the comparison. If one accepts that the use of the retro-cue and the post-cue represents a way to experimentally discriminate between sensory memory and working memory, then a parsimonious interpretation of the present and previous results on this topic is that sensory memories as well as working memory depend on attention, even though to a different extent (as indicated by the difference in attentional benefits between iconic and working memory condition), and perhaps through the modulation of different phases or cognitive operations. Consistent with this conclusion, Souza and Oberauer [8] proposed an elegant theory whereby representations can vary on a continuum of robustness and plausibly in a single working memory store. Attention to a representation during maintenance elicited by retro-cues would further increase its robustness, therefore protecting it from visual interference. According to this proposal, retro-cues would not assess a different memory structure such as fragile visual short-term memory.

### Exogenous vs. endogenous selective spatial attention

When considering the role of attention in the different phases of memory, from encoding to conscious retrieval, it is worth considering the distinction between endogenous and exogenous attention. Indeed, we found important differences between exogenous and endogenous attentional orienting regarding their modulation over the different memory stages. Accumulative evidence shows that attention endogenously controlled by top-down signals yields qualitatively different effects from exogenously captured attention on various aspects of information processing, such as perception [14–17], spatial conflict resolution [18], visual sensory memory [21] and visual working memory [19,20]. This suggests that endogenous and exogenous attentional mechanisms constitute at least in part two independent systems. This hypothesis is also supported by neurostimulation studies, as well as neuropsychological and animal studies, which have pointed out that endogenous and exogenous attention are implemented by partially segregated brain networks [12], or are characterized by different dynamics of activation of overlapping networks [23]. In this vein, we found that while endogenous attentional bias increases through the three stages of short-term memory, exogenous attentional bias triggered by task-irrelevant peripheral cues remains constant. Furthermore, while accuracy at the endogenous focus was comparable across the three memory stages, it progressively decreased through memory stages at the exogenous focus, exactly as in the unattended locations and in the control condition. This finding indicates that exogenously attending a single item does increase its probability of being remembered, but it does not totally protect it from interference and trace decay, at variance with what occurs, instead, for endogenously attended items.

A possible way to interpret this difference is that purely exogenous cues might only affect visual *selection* and encoding, while endogenous attention might be also associated with *consolidation* and maintenance in memory, as proposed in the attentional blink literature [33]. More specifically, exogenous cues might boost the representation of the precued item, but only for a short delay after which its activation would start to decay and be less robust to visual interference. On the other hand, endogenous cues would contribute not only to select the precued item, but also to consolidate it in working memory thus maintaining its representation at maximal levels for a longer delay, and protecting it from interference and natural decay. In other words, endogenous cues, but not exogenous cues, would contribute to consolidation into working memory. However, it must be noted that the different data pattern at valid endogenously vs. exogenously attended locations have been drawn from different experiments. Thus, these conclusions should be considered with caution. Further studies combining both attentional manipulations simultaneously and/or as a within-participant factor are definitely required to assess this conclusion.

### Is the sensory memory content P-conscious or A-conscious?

As described in the Introduction, according to Lamme [9] the content of sensory memories reflects phenomenally conscious visual representations implemented by local recurrent activations between visual areas. If so, then our findings clearly indicate that phenomenal consciousness is also modulated by selective attention. However, the above-mentioned parallelism between sensory memory and phenomenal consciousness is anything but universally accepted. For example, Naccache [34], analysing the seminal Sperling experiment’s results, questioned the supposed equation between sensory memory and phenomenal consciousness and advanced a radical proposal according to which the P-conscious content is nothing else but a sub-set of A-conscious content. According to Naccache [34] when facing with attention a visual memory array of letters, each individual letter of the array is *unconsciously* represented in the ventral cortical visual pathway while, at the same time, the representation of the global background, providing spatial information about items location and the general array structure, is plausibly also coded in the dorsal visual pathway. After approximately 300 ms, the participant would consciously access a visual representation that integrates the global background description computed by the dorsal network with the precise identity of a subset of letters (the attended ones) implemented by the ventral network. However, this happens only if these two pieces of information converge in the fronto-parietal activity reverberation, which is associated with top-down attentional modulations and working memory. The integration of the ventral and dorsal visual unconscious representations in the integrated A-conscious representation would also include a sort of filling-in mechanism that would complete the imprecise information about the identity of the unattended items by using the precise identity information of the focused ones (see also Kouider et al. [2]), thus explaining the (illusory?) sensation of rich and detailed perception associated with iconic memory. Consistent with these claims, neuroimaging evidence has shown coupled activity of fronto-parietal networks, including the right inferior parietal lobule and the left frontal eye field, when attentional cues facilitated the conscious perception and localization of near-threshold targets [35]. Networks including the right parieto-temporal region could thus be essential to the attention-based integration of perceptual identity and spatial localization; their damage often results in striking inattention and unawareness for left-sided objects (visual neglect; see Bartolomeo [36]), or a wrong matching of locations with objects’ features in memory [37].

Ultimately, the above-mentioned debate between P-consciousness theorists (like Lamme [4,9]), and A-Consciousness theorists (like Naccache [34]) concerning the association between sensory memory and phenomenal consciousness, regards the role that is attributed to attention in the interpretation of Sperling’s iconic memory experiment. P-consciousness theorists consider attention as being essential for A-consciousness (whose content is supposed to be stored in working memory), but not for P-consciousness (whose content is supposed to be stored in sensory memory); whereas A-consciousness theorists contend that attention represents a necessary condition for A-consciousness, which would be the only possible kind of (reportable) consciousness, so that without attention even the content of iconic memory would have no access to consciousness.

In our view there are at least two phases of the Sperling experiment in which the role of attention is particularly clear. Firstly, in a typical Sperling paradigm, participants need to focus their attention on the spatial region where the to-be-remembered stimuli will be presented. Even in the absence of precues (as in the typical task), the area of the screen where the letters will be presented needs to be selected, prioritizing task-relevant and inhibiting task-irrelevant information. In support of this view, the addition of a double, competing task, can decrease both iconic memory [11,31,32] and fragile memory [7]. Our data constitute further proof that selective attention exerts an important role in sensory memory, at least during encoding, by showing that the distribution of attention to task-relevant locations produces a clear modulation of its content. Moreover, once the stimuli have been encoded, the task execution requires to process the retro-cue and to orient the attentional focus towards the indicated location. This means that the very assessment of sensory memories is impossible without assuming that selective attention plays a role both during the encoding phase, and in the use of the retro-cue.

According to the traditional interpretation, however, participants are able to recall the letters contained in virtually any retro-cued locations; thus, all the letters presented in the uncued locations are considered to be available right after the array presentation in the sensory memory store. This view, which is the one supported by P-consciousness theorists, conceptualizes the attentional focusing associated with retro-cue as a mere tool to read out, without any modulation over the representations implemented in the early visual areas. In other words, P-consciousness theorists consider that sensory memory traces are maintained active independently of attention. However, recent studies (see Souza & Oberauer [8] for a review) suggest that when attention is allocated in a controlled fashion to the individual representations of visual short-term memory through the retro-cues (visual working memory in their words), it flexibly modulates them by strengthening the representations of the individual items, stabilizing them against perceptual interference and selecting them for retrieval. In other words, the retro-cue, more than simply being a “reader” of the memory representation, would produce a modulation/modification of the representation itself. Thus, the reportable content of short-term memory would be the result of the interactions with focal attention elicited by the retro-cue. Consistent with this possibility, Sergent et al. [38] demonstrated that retro-cues can bring to conscious perception a masked target which would otherwise have gone undetected. Remarkably, this can occur for retro-cues presented hundreds of milliseconds after target disappearance. This result represents a strong clue that what is measured in sensory memory tasks is the by-product of the indissoluble interaction between attention and the unknowable (therefore unconscious) content of the assumed sensory memory representations.

According to our theoretical point of view, and in line with the global workspace model [39], sensory memory and working memory *reported* representations are implemented by the reverberation of activation between visual and fronto-parietal regions. In both cases we assume that the attentional modulation of these representations can be implemented by incrementing the activity between posterior and fronto-parietal regions during the encoding phase (that is before and during the stimuli presentation) and/or during maintenance (once the stimuli have disappeared). On the basis of Buschman and Miller results [23], the global reverberation may be modulated by increasing the activity of posterior regions and then, consequently, that of fronto-parietal regions as it happens with exogenous attentional capture, or by increasing fronto-parietal activity and then, consequently, posterior regions activity by means of endogenous attentional control.

During encoding, in both the retro-cue condition (iconic and fragile memory) and the post-cue condition (working memory), participants do not know in advance which stimulus will be probed; they will thus be likely to endogenously distribute their attention among all the items. However, during maintenance, while in the post-cue condition (working memory condition) attentional resources are still distributed among items, in the retro-cue condition (iconic and fragile memory conditions) attentional resources are polarized on one specific element once the retro-cue is presented. For this reason, less attentional resources are voluntary deployed to the probed element in the post-cue condition than in the retro-cued condition. If that is true, then the main difference between sensory memories and working memory, could be conceptualized as the difference of the total endogenous attentional resources allocated to the probed item during encoding and maintenance, which could be reflected in a modulation of the intensity or the stability of the global reverberation between ventral sensory regions and fronto-parietal networks: a stimulus (or a change in the stimulus) will be reported if the specific reverberation that represents it exceeds an intensity or a temporal threshold. Probed items in sensory memory conditions would be just more suitable to exceed these thresholds than items in working memory conditions, because the fronto-parietal activity associated with their processing is reignited by the retro-cue. Endogenous precues would basically do what retro-cues do, but before the stimuli are presented: they would contribute to maintain active and consolidate the to be-probed representation over time facilitating it in the internal competition among the items of the memory array and in the external competition with new incoming information (i.e. the masking effect). Exogenous precues would instead increase the selection mechanism by temporarily boosting sensory activity. However, this increase of activation would be short-lived, thus mainly facilitating sensory memory rather than working memory, as indicated by the reduction of accuracy at exogenously cued locations that we observed in working memory compared with iconic memory (Experiment 2).

## Conclusions

Our results indicate that the modulation of selective spatial attention during encoding clearly affects the three stages of visual short-term memory. We also observed that when attention is voluntarily directed by endogenous cues, attentional benefits increased from iconic to working memory, boosting up the general attentional effect. Attentional capture by purely exogenous cues triggered a general attentional effect, which was comparable across the three short-term memory stages. The differences between endogenous and exogenous modulations of the three memory stages can be interpreted in terms of selection and maintenance mechanisms. Specifically, automatic attentional capture might only affect visual encoding and selection, boosting up the representation of the attended item, but only for a short time, after which its activation would start to decay and be less robust to visual interference. On the other hand, endogenous control of attention would contribute not only to select the item but also to consolidate it into working memory, thus maintaining its representation strong over time, and protecting it from interference and natural decay.
